# Data-Driven Identification of Stroke through Machine Learning Applied to Complexity Metrics in Multimodal Electromyography and Kinematics

**DOI:** 10.3390/e26070578

**Published:** 2024-07-07

**Authors:** Francesco Romano, Damiano Formenti, Daniela Cardone, Emanuele Francesco Russo, Paolo Castiglioni, Giampiero Merati, Arcangelo Merla, David Perpetuini

**Affiliations:** 1 Department of Engineering and Geology, University G. D’Annunzio of Chieti-Pescara, 65127 Pescara, Italy; francesco.romano005@studenti.unich.it (F.R.); d.cardone@unich.it (D.C.); arcangelo.merla@unich.it (A.M.); 2 Department of Biotechnology and Life Sciences, University of Insubria, 21100 Varese, Italy; damiano.formenti@uninsubria.it (D.F.); paolo.castiglioni@uninsubria.it (P.C.); giampiero.merati@uninsubria.it (G.M.); 3 Padre Pio Foundation and Rehabilitation Centers, 71013 Foggia, Italy; sic@centripadrepio.it; 4 IRCCS Fondazione Don Carlo Gnocchi, 20148 Milan, Italy; 5 UdA-TechLab, University G. D’Annunzio of Chieti-Pescara, 66100 Chieti, Italy

**Keywords:** ischemic stroke, motor dysfunction, EMG, gait analysis, machine learning, complexity analysis, sample entropy, approximate entropy, spectral entropy, conditional entropy

## Abstract

A stroke represents a significant medical condition characterized by the sudden interruption of blood flow to the brain, leading to cellular damage or death. The impact of stroke on individuals can vary from mild impairments to severe disability. Treatment for stroke often focuses on gait rehabilitation. Notably, assessing muscle activation and kinematics patterns using electromyography (EMG) and stereophotogrammetry, respectively, during walking can provide information regarding pathological gait conditions. The concurrent measurement of EMG and kinematics can help in understanding disfunction in the contribution of specific muscles to different phases of gait. To this aim, complexity metrics (e.g., sample entropy; approximate entropy; spectral entropy) applied to EMG and kinematics have been demonstrated to be effective in identifying abnormal conditions. Moreover, the conditional entropy between EMG and kinematics can identify the relationship between gait data and muscle activation patterns. This study aims to utilize several machine learning classifiers to distinguish individuals with stroke from healthy controls based on kinematics and EMG complexity measures. The cubic support vector machine applied to EMG metrics delivered the best classification results reaching 99.85% of accuracy. This method could assist clinicians in monitoring the recovery of motor impairments for stroke patients.

## 1. Introduction

A stroke is a remarkable medical condition consisting of the sudden and unexpected interruption of the blood flow to the brain, leading to cell damage and tissue necrosis in worse instances [1]. According to the World Stroke Organization (WSO) Global Stroke Fact Sheet 2022, stroke is the second leading cause of death worldwide and the third-leading combined cause of death and disability worldwide, with over 12.2 million new strokes each year [2]. In addition, stroke survivors may develop complications such as paresis or hemiparesis. These movement impairments can make it challenging to carry out daily activities which requires balance and walking. As a result, patients may require various treatments, including robotic rehabilitation, biofeedback training rehabilitation, motor imagery paradigms, and peripheral electric stimulation. These treatments play a crucial role in improving or partially restoring locomotor functions [3,4,5,6]. Gait analysis can be used to assess muscular activity, coordination, and gait patterns in stroke hemiparetic subjects, to provide them the best personalized rehabilitation.

Gait analysis offers significant insights on anomalies in movement and functional limitations, usually based on two main types of approaches: quantitative measurements using instrumented gait analysis (IGA) and observational evaluations using video gait analysis (VGA) [7]. Instrumented gait analysis utilizes a range of instruments and technologies to gather accurate and unbiased measurements of movement patterns while walking. These measurements include factors such as body motion, exerted forces, electrical muscle activity, foot pressure distribution, and metabolic activation [8,9].

Gait analysis involves the collection of dynamic electromyography (EMG) data to assess muscle activation patterns while walking. This allows for the examination of muscle function and coordination [10]. Of note, surface electromyography (sEMG), applied to the skin over the muscles, has been employed for a long time in gait analysis applications [11], thanks to its non-invasiveness. Specifically, EMG refers to the process of quantifying the electrical impulses generated by muscles that are in an active state [12]. In detail, when a motor neuron stimulates a muscle fiber, the ionic concentration around the lipid membrane changes. This leads to the creation of a gradient of electrical potential known as a biopotential. The EMG sensors detect the concurrent presence of these potentials generated by muscle activation, which represents the combined motor unit action potential. This approach is often used to examine potential pathological alterations [13,14] or rehabilitative advantages [15], as well as to assess neuromuscular responses in sports contexts [16,17]. Especially, the implementation of EMG signal analysis techniques seems to play a relevant role in robotic gait training rehabilitation [18]. 

Moreover, instrumented gait analysis may use accelerometry and inertial sensor-based systems to evaluate spatiotemporal gait parameters and lower limbs typical angles, hence improving the assessment of gait features [19,20,21]. 

Among the other technologies able to assess walking patterns in a medical setting, stereophotogrammetry is a sophisticated imaging method used to precisely evaluate human movement patterns [22]. Stereophotogrammetry is generally acknowledged as the most reliable technique for analyzing human movement, offering accurate data for biomechanical research and clinical gait analysis [23]. This technique utilizes 3D optical camera systems and skin markers to record and examine movement data, allowing for the measurement of several kinematic parameters while walking [22].

Stereophotogrammetry may be used to accurately monitor joint movements, muscle activity, and other important factors during walking, provides a comprehensive analysis of gait characteristics [24,25,26].

Gait analysis using both EMG and stereophotogrammetry may therefore provide comprehensive gait profiles by combining kinematic and muscle activity data, allowing for an accurate characterization of gait abnormalities [27]. 

Data analysis plays a crucial role in increasing the potential of gait analysis to discriminate healthy controls (HC) from pathological patients. In this perspective, defining proper metrics able to identify abnormalities in the gait pattern is fundamental. So far, complexity metrics have been proved to be highly suitable for identifying abnormal gait conditions when applied to stereophotogrammetric and EMG data. Among the complexity measures available, the entropy, in the context of information theory and consequently in the context of signal analysis, provides insights into the irregularity, unpredictability, and complexity of a signal. Particularly, by quantifying the entropy of EMG and kinematic data, it is possible to capture essential characteristics of muscle activation patterns and gait dynamics in stroke hemiparetic subjects. The ability of complexity metrics to identify stroke patients could be increased by employing machine learning (ML), a subset of artificial intelligence (AI) that focuses on the development of algorithms and statistical models for classification and regression purposes.

The aim of this study is to investigate the capability of ML classifiers applied to complexity metrics of EMG and kinematic signals to differentiate stroke patients (SP) from age-matched healthy controls (HC). Specifically, complexity measures such as approximate entropy, sample entropy, fuzzy entropy, spectral entropy and conditional entropy, were employed to analyze both EMG and gait signals. In particular, approximate entropy and sample entropy were included to assess the regularity or predictability of a signal, reflecting the stability or complexity of muscular activity or gait patterns. On the other hand, fuzzy entropy extends the concept of entropy measures by considering uncertainty in fuzzy data, whereas spectral entropy provides information about its spectral complexity revealing patterns of muscle activation or movement dynamics across frequency domain. Finally, conditional entropy measures the amount of complexity of a time-series signal given the knowledge of another, proving to be a valuable tool to estimate the interdependence between EMG and kinematic signals. 

## 2. Materials and Methods

### 2.1. Experimental Procedure and Data Acquisition

The study utilized a publicly available dataset provided by Van Criekinge et al. [28]. This dataset includes biomechanical data from 138 able-bodied individuals (65 males and 73 females) aged 21 to 86 years and 50 stroke survivors (34 males and 16 females) aged 19 to 85 years. The SP group was composed of 39 hemorrhagic and 11 ischemic stroke patients. Participants walked barefoot at their preferred pace. Criteria for inclusion in the stroke survivor group included diagnosis of either hemorrhagic or ischemic stroke confirmed by computerized tomography or magnetic resonance imaging, no prior history of stroke, stroke occurring within the last five months, and age between 18 and 85 years. Relevant medical information, including stroke diagnosis, medical history, and stroke onset details, was obtained from medical records. The dataset includes kinematic and sEMG data. As the analysis of gate and EMG alone are not sensitive enough to distinguish between hemorrhagic and ischemic causes, data were treated regardless of the cause of the stroke. In addition, it should be highlighted that the limited numerosity of the hemorrhagic and ischemic groups could impair the performance of ML algorithms, resulting in not reliable classifications if considering the hemorrhagic and ischemic patients separately. Kinematic signals were recorded using a three-dimensional passive motion capture system consisting of 8 Vicon T10 cameras (©Vicon Motion System Ltd., Oxford, UK) capturing at 100 frames per second with a resolution of 1 megapixel (1120 × 896). EMG data were collected using a synchronized 16-channel telemetric wireless surface system (©Zerowire, Cometa, Barregin, Italy). For further details about the experimental protocol and data preprocessing are reported in Van Criekinge et al. [28].

### 2.2. Data Processing

The database contains preprocessed data acquired at a sample frequency of 1000 Hz, ready for subsequent analysis. Specifically, for gait data analysis, calcaneus coordinates were employed, in order to calculate the following parameters: approximate entropy (ApEn), sample entropy (SampEn), fuzzy entropy (FuzzyEn), spectral entropy (SpEn), and conditional entropy of gait signal given the knowledge of the EMG data (CondEn). In terms of EMG signal analysis, data from the gastrocnemius muscle were examined, and same entropy features were evaluated, with the addition of a multi-scale approach employed on ApEn, SampEn and FuzzyEn [29], to investigate complexity across different time-scales. Precisely, starting from an EMG signal with a size of 2000 samples, equivalent to two subsequent strides, a down-sampling procedure was employed to obtain a new set of subsampled signals, by letting apart an n number of subsequent samples every time a sample is taken, where n in 1,…,20, resulting in a 20-signal set, where the first one is equivalent to the original one, and the twentieth is the most coarse grained data, corresponding to a size of 100 samples. Notably, entropy metrics were estimated for the right side of the body in able-bodied subjects and for the paretic side in hemiparetic subjects.

Regarding the determination of the optimal parameters for ApEn, SampEn, FuzzyEn, and CondEn, there are no specific guidelines available. However, for biological signals, certain values are commonly used as they are appropriate for the dynamics of physiological signals [30]. The recommended approach is to utilize an embedding dimension that spans from 1 to 3. Crucially, the decision may be influenced by the duration of the signal to be analyzed. Specifically, the time series should ideally range between 10^m^ and 20^m^ in length. Thus, when performing a multiscale procedure, it is important to consider the length of the coarse-grained time series [29,31]. Specifically, the number of samples in the coarse-grained series should be greater than 10^m^ to 20^m^, and the new sample frequency should be higher than the frequencies of the signal’s harmonics [32]. The tolerance factor, denoted as *r*, is often expressed as a percentage of the standard deviation of the time series, and typical options range from 0.1 to 0.25 times the standard deviation [33].

### 2.3. Entropy Measures

In information theory, Shannon entropy *H*(*X*) is a measure of the randomness uncertainty associated with a random variable *X* of length N≥1 such as $X=x_{1},\;x_{2},\ldots ,x_{N}$. Specifically, entropy quantify the average amount of information (bit) needed to describe the outcome of *X*, where *H*(*X*) = 0 bits represent the minimum value of information needed to describe the outcome that arise when the probability distribution is completely deterministic, meaning there is no uncertainty in the variable’s outcome, while $H\left(X\right)=\log_{2}N$ bits represent the maximum value of information needed which occurs when all possible outcomes have the same probability to occur. Mathematically, entropy in defined as

$$H\left(X\right)=-\sum_{i=1}^{N}p\log_{2}p(x_{i})$$

where $p\left(x_{i}\right)$ are probabilities of occurrence of each possible outcome. Over time, numerous adaptations of this concept have surfaced to meet evolving demands in real-world use, notably in fields such as time-series analysis. 

#### 2.3.1. Approximate Entropy

ApEn [34] was introduced for the first time to quantify regularity of biological time-series, without taking into account knowledge about the system that the data come from. The main idea is that biological time series, which come from more ordered and complex systems such as biological systems, are often characterized by repetitive templates. ApEn evaluates the negative natural logarithm of the conditional probability that short templates of length m points are repeated during the whole time series [34]. Firstly, the vectors from the time series are organized in the following form, called the pseudo-phase [35]:

$$y\left(i\right)=[x\left(i\right),\ldots ,x\left(i+m-1\right)]$$

$$y\left(j\right)=[x\left(j\right),\ldots ,x\left(j+m-1\right)]$$

Approximate entropy can be defined as

$$ApEn=\Phi^{m}(r)-\Phi^{m+1}(r)$$

where *m* is the embedding dimension, *r* the tolerance level and *Φ(r)^m^* is the probability that two subsets from the signal match for m data points:

$${\Phi (r)}^{m}=\frac{1}{N-m+1}\sum_{i=1}^{N-m+1}lnC_{i}^{m}(r)$$

where

$$C_{i}^{m}\left(r\right)=\frac{Number\;of\;vector\;pairs\;such\;that\;d<r}{N-m+1}$$

where *d* represents the Euclidean distance between points. Similarly, *Φ^m+^*^1^*(r)* is evaluated for *m* + 1 data points. Additionally, ApEn metric was embraced due to its capability in the analysis of signals characterized by noise or fluctuations such as EMG signals and to its sensitivity to discern patterns over both short-term and long-term durations [34]. In this study, ApEn was assessed for *r* = 20% of the standard deviation of the signal and *m* = 2, for both EMG and kinematic signals.

#### 2.3.2. Sample Entropy

SampEn [30] was proposed as an alternative to ApEn aiming to overcome certain practical issues. Unlike ApEn, SampEn does not consider self-matches between vectors and exhibits a reduced dependence on record length, making the measure more solid and precise, particularly when relatively short time-series data, such as EMG and kinematic signals, are examined [30]. SampEn is defined as

$$SampEn=ln\frac{\Phi^{m}(r)}{\Phi^{m+1}(r)}$$

where $\Phi^{m}(r)$:

$$\Phi^{m}\left(r\right)=\frac{1}{N-m}\sum_{i=1}^{N-m}lnC_{i}^{m}(r)$$

Comparably, *Φ^m+^*^1^*(r)* is expressed for an embedding dimension of *m +* 1. SampEn metric was assessed in this study considering *r* = 20% of the standard deviation of the signal and *m =* 2, for both EMG and kinematic data.

#### 2.3.3. Fuzzy Entropy

FuzzyEn is a complexity measure, used the analysis of fuzzy time series data, incorporating concepts from fuzzy logic. By definition, a fuzzy set encompasses elements with varying membership degrees, as opposed to classical sets where elements possess complete membership [36,37]. To compute FuzzyEn, a fuzzy membership function is employed to assigns real values to elements of a fuzzy set within the interval [0, 1], reflecting varying degrees of membership. Firstly, the vectors from the time series are organized in the following form, called the pseudo-phase:

$$\bar{y}\left(i\right)=[x\left(i\right)-\bar{x}\left(i\right),\ldots ,x\left(i+m-1\right)-\bar{x}\left(i\right)]$$

$$\bar{y}\left(j\right)=[x\left(j\right)-\bar{x}\left(j\right),\ldots ,x\left(j+m-1\right)-\bar{x}\left(j\right)]$$

with $\bar{x}(i)$ the mean value of *y(i)*, FuzzyEn is defined [15] as

$$FuzzyEn=ln\frac{\Phi^{m}}{\Phi^{m+1}}$$

where

$$\Phi^{m}=\frac{1}{N-m}\overset{N-m}{\underset{i=1}{\sum }}\overset{N-m}{\underset{j=1,j\neq i}{\sum }}\frac{D_{i,j}^{m}}{N-m-1}$$

where

$$D_{i,j}^{m}=\mu \left(d\left({\bar{y}}_{i}^{m},{\bar{y}}_{j}^{m}\right)\right)$$

is the fuzzy membership matrix, and *μ(x)* is the fuzzy membership function that leads to a diminished impact of the threshold parameter choice:

$$\mu (x)=e^{-(\frac{y}{r}{)}^{n}}$$

FuzzyEn was incorporated as a feature due to its consistency, particularly in scenarios involving noisy signals characterized by low amplitude parameters, such as EMG. 

#### 2.3.4. Spectral Entropy

SpEn measures the uncertainty about an outcome at a certain frequency or in a frequency band, quantifying the spectral complexity of the time-series signal [38,39]. SpEn is defined [39] as

$$SpEn=-\overset{N}{\underset{i=1}{\sum }}p_{i}ln(p_{i})$$

where $p_{i}$ represents the power spectral density distribution obtained from power spectral density P^(ωi), such as

pi=P^(ωi)∑i=1NP^(ωi)

Importantly, given that SpEn yields a vector as its output, statistical descriptors including mean, standard deviation, skewness, and kurtosis were extracted as features. Importantly, only for this metric the first four moments of a distribution were extracted.

#### 2.3.5. Conditional Entropy

CondEn extends the concept of entropy to situations in which information about another variable is available, measuring the amount information needed to describe the outcome of a random variable Y when another random variable X is known. CondEn is defined as the expected value *E* of the entropy of the conditional distribution p(Y|X), averaged over the conditioning random variable [40]:

$$CondEn=-\underset{x\in X}{\sum }\underset{y\in Y}{\sum }p(x,y)log_{2}p(y|x)=-Elog_{2}p(Y|X)$$

where p(x,y) and p(y|x) are respectively the joint probability distribution and the conditional probability distribution. The hypothesis is based on the idea that muscle activity during walking significantly influences gait patterns, thereby indicating a strong interdependence between EMG and kinematic data. As a result, CondEn emerged as a pivotal metric to unveil the interdependencies between EMG and kinematic data. 

### 2.4. Statistical Analysis

Several classification models were employed to discern SP from HC, aiming to evaluate classification efficacy across diverse classifier types. The considered models are coarse tree, logistic regression, kernel naive Bayes, cubic SVM, fine kNN, bagged trees, and medium neural network (medium NN). Precisely, the models utilized for the task were trained using the EMG and kinematic complexity parameters separately, and subsequently combined, considering the CondEn metric as well. This approach allowed a comparative analysis of the classification performance attained through the employment of unimodal EMG and kinematic features versus their combination. Further, given the imbalanced nature of the two classes, consisting of 138 HC and 50 SP, an iterative approach was employed to ensure class balance and mitigate potential overfitting effects related to participant allocation within the fold used to train and test the models. Specifically, the class sizes were determined based on the smaller class (i.e., 50 samples), with elements from the larger class randomly selected in an iterative manner across 1000 iterations, enabling the exploration of all possible sample combinations. Importantly, due to the high feature dimensionality with respect to the class sizes (i.e., SP and HC), feature selection was conducted using the minimum-redundancy maximum-relevance method (MRMR) during the cross-validation framework. Significantly, the MRMR algorithm takes advantage of the output labels and generates data overfitting without separating samples in the train and test sets. Therefore, just as the hyperparameter optimization of the various classifiers, the feature selection framework was repeatedly performed within the nested cross-validation (nCV). In order to enhance the hyperparameters and guarantee the model’s capacity to generalize without overfitting, it is important to have three separate datasets. Initially, a training set is employed to train the model using different hyperparameter values. Additionally, a validation set is utilized to determine the optimal hyperparameters based on performance. Ultimately, a test set is employed to assess the ultimate performance of the final model. This methodology enables efficient optimization of hyperparameters and impartial evaluation of the model’s performance on unobserved data. However, if there is a smaller number of samples available, this data separation could significantly decrease the size of the training sample, making it challenging for the data-driven model to be accurately fitted. The nCV is a technique that extends the aforementioned procedure in order to mitigate the negative effects of sample loss over several sets, preventing biases in the results and avoiding overfitting of the data [41,42]. Specifically, the data are divided into folds, and the model is trained in a nested manner on all but one fold of the data. The inner loop determines the best hyperparameters (specifically, it selects features using the MRMR approach) for validation purposes. Meanwhile, the outer loop evaluates the model’s performance throughout multiple iterations for testing. This work employed a 10-fold nCV approach to train, validate, and test several models. In order to determine the best model hyperparameter and identify the chosen features mentioned in the manuscript, a majority voting approach was utilized during the cross-validation cycles. 

The first model that was trained was the coarse tree model. The minimum leaf size (MinLeaf) and the maximum number of splits (MaxNumSplits) were considered using 1–39 and 1–77 as ranges for optimization, respectively. The criterion used to choose the best split at each node was Gini’s diversity index (GDI). The second model was a logistic regression, where the regularization parameter was taken into consideration for optimization, using 30 learning cycles and Lasso as regularization technique. Furthermore, a kernel naive Bayes model was tested, examining the bandwidth (h) for the hyperparameter optimization. In addition, for the cubic SVM, the box constraint (C) and the kernel scale (γ) were optimized, and for the fine kNN, the number of neighbors (k) was optimized considering the Euclidean distance and the same weight for all the neighbors.

Moreover, a bagged trees model was a bagging ensemble of decision trees (bagged trees). This model exploits several decision trees, each trained on a random subset of the training data. In this study, the number of trees (NumTrees), MinLeaf, and MaxNumSplits were taken into account. The ranges considered for hyperparameter optimization were 5–100 for NumTrees (with a step of 10), 1–39 for MinLeaf, and 1–77 for MaxNumSplits. A bagging procedure randomly selected a subset of the training data for each tree, repeating the process for 30 learning cycles, ensuring sufficient diversity among trees in the ensemble. Finally, a medium neural network (medium NN) was defined, considering the number of layers (range: 1–3), the size of the layers (range: 1–300), the regularization parameter (range: 1.27 × 10^−^^7^–1.28 × 10^3^) as hyperparameters to be optimized. The activation function was the rectified linear unit activation function, and the maximum number of iterations was set to 1000.

Performance evaluation of the classifiers involved sensitivity, specificity and accuracy metrics, derived from the relative confusion matrix. Moreover, the receiver operating characteristic (ROC) were computed for each model. Furthermore, the DeLong test was conducted in order to compare the performance of the model across the three different features set. The DeLong test is utilized to compare the area under the curve (AUC) values of two or more ROC curves that are correlated, typically originating from the same group of individuals [43]. The test takes into account the correlation between the ROC curves, which is crucial as employing the same dataset for several models or tests results in correlated ROC curves. In order to conduct the DeLong test, it is imperative to compute the AUC for each ROC curve and represent them as a U-statistic. This U-statistic is determined by comparing the predicted scores between different classes. After, it is necessary to calculate the variability of the AUC and the correlation between the AUCs of two associated ROC curves. Finally, the difference in AUCs is normalized by the expected standard deviation to create a Z-test statistic. The *p*-value is derived from the standard normal distribution. Additionally, an independent samples *t*-test was employed to investigate group differences (SP vs. HC) between the metrics selected by the MRMR approach. A *p*-value lower than 0.05 was considered statistically significant. The entire analysis was conducted in MATLAB R2023b (MathWorks, Inc., Natick, MA, USA). 

## 3. Results

The trained models effectively evaluated the motor impairments linked to stroke, employing either unimodal kinematic, EMG, and multimodal kinematic + EMG, as shown in Table 1.

Concerning the kinematic models, the optimized MinLeaf and MaxNumSplits for the coarse tree model were 6 and 2, respectively. For the logistic regression, the regularization parameter was optimized as 5.636 × 10^−^^6^. Concerning the kernel naive Bayes model, h was 0.267, whereas for the cubic SVM, C and γ were 136.55 and 8.705, respectively. The number of neighbors for the kNN classifier was 7, and for the bagged trees model, NumTrees = 20, MinLeaf = 2, and MaxNumSplits = 75 were obtained. For the medium NN, the number of layers was 2, the size of the layers was 25, and the regularization parameter was 6.932 × 10^−^^7^.

As far as it concerns the EMG models, MinLeaf = 13 and MaxNumSplits = 14 were obtained for the coarse tree model. Regarding the logistic regression, the optimized regularization parameter was 3.696 × 10^−^^6^, whereas h = 0.070 was found for the kernel naive Bayes model. Concerning the cubic SVM, C = 996.44 and γ = 24.302 were obtained. For the kNN, k = 2 was the optimized number of neighbors, and for the bagged trees model, NumTrees = 70, MinLeaf = 2, and MaxNumSplits = 17 were delivered. For the medium NN, the number of layers was 2, the size of the layers was 25, and the regularization parameter was 1.465 × 10^−^^7^.

Regarding the kinematic + EMG models, for the coarse tree model, the MinLeaf was 10 and MaxNumSplits was 4. The regularization parameter of the logistic regression was 5.622 × 10^−^^6^. Concerning the kernel naive Bayes model, h was 0.275, and for the cubic SVM, C was 994.51 and γ was 6.586. The number of neighbors for the kNN classifier was estimated as k = 6. For the bagged trees model, NumTrees = 60, MinLeaf = 2, and MaxNumSplits = 5. For the medium NN, the number of layers was 2, the size of the layers was 25, and the regularization parameter was 2.397 × 10^−^^4^.

**Figure 1:**
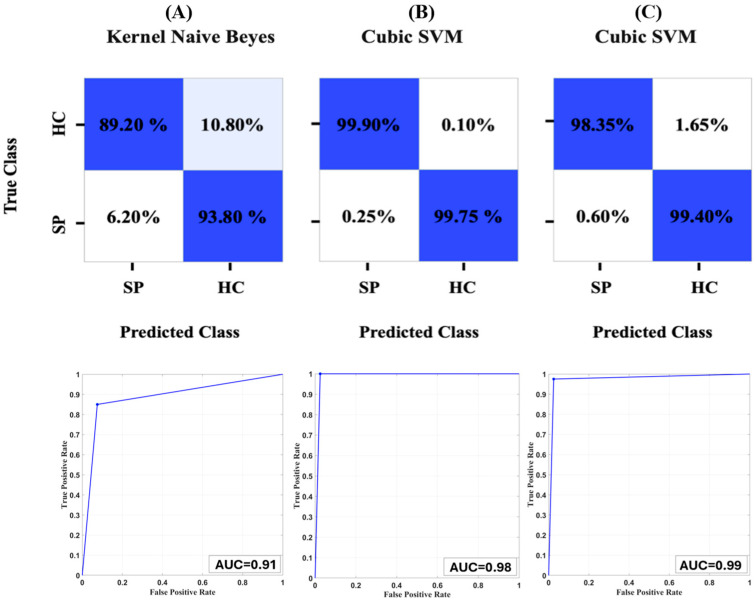
Confusion matrix associated with the performance of the models fed with (**A**) unimodal kinematic metrics, (**B**) unimodal EMG parameters, and (**C**) kinematic + EMG parameters.

Figure 1 shows the confusion matrices associated with the most performing model, in term of accuracy, fed with the unimodal kinematic metrics set (Figure 1A), unimodal EMG metrics set (Figure 1B), and multimodal kinematic + EMG (Figure 1C).

Concerning the MRMR procedure, the input features selected when feeding the classifiers with only the kinematic parameters set are X axis ApEn, Z axis ApEn, X axis SampEn, Y axis SampEn, Z axis SampEn, X axis SpEn mean, X axis SpEn kurtosis (kurt), Y axis SpEn mean value (mean), X axis FuzzyEn, and Z axis FuzzyEn. Regarding the models fed with the EMG parameter set, the selected features by MRMR are ApEn, SampEn, SpEn standard deviation (std), SpEn kurt, SpEn skew, FuzzyEn, multiscale ApEn (*n* = 2), multiscale ApEn (*n* = 15), multiscale FuzzyEn (*n* = 5). In conclusion, the features selected for the kinematic + EMG + CondEn are X axis ApEn, X axis SampEn, Z axis SampEn, SpEn std (EMG), CondEn EMG-X axis gait, CondEn EMG-Y axis gait, CondEn EMG-Z axis, multiscale ApEn (*n* = 16), and multiscale FuzzyEn (*n* = 8). The unpaired t-tests between the metrics selected by the MRMR procedure between the two groups are reported in Table 2.

Additionally, the results of the DeLong test comparing the AUC of the ROC curves of the different models are reported in Table 3.

## 4. Discussion

This study investigates the capability to identify gait disorders related to stroke through ML approaches applied to complexity measures obtained from kinematic and EMG signals. The results demonstrated that EMG metrics-based models exhibited a significantly better capability in the classification of post-stroke individuals than kinematic metrics-based models. Specifically, the best result was obtained by a Cubic SVM fed with EMG complexity features, reaching an accuracy of 99.85%. Conversely, the models fed with gait complexity features showed reduced ability, reaching a maximal result of 90.26% of accuracy, obtained with a kernel naive Bayes model. Moreover, the models fed with multimodal kinematic and EMG entropy features have shown optimal results, reaching the maximal accuracy of 98.98% implementing a cubic SVM. 

A feature selection was performed using the MRMR algorithm. Almost all the features selected exhibited a significant difference between the two groups. Specifically, the kurtosis of the SpEn evaluated on the X direction and the mean value of the SpEn computed on the Y axis were not statistically different between the two groups, whereas the mean value of the SpEn computed on the X axis showed differences between SP and HC. Notably, SpEn is a measure of the complexity or unpredictability of a signal in the frequency domain. It provides information about how the power of a signal is distributed across different-frequency components. Higher spectral entropy indicates a more complex or less predictable signal. Concerning the statistical metrics evaluated on SpEn, the kurtosis describes the shape of a distribution’s tails in relation to its overall shape (high kurtosis indicates heavy tails, while low kurtosis indicates light tails), and the mean value is a central tendency descriptor. Hence, the no significant differences evaluated for these metrics could be related to similarities in the movement patterns in the forward–backward motion and in the vertical direction. These similarities can result from compensatory mechanisms in SP, where they adapt their gait to resemble that of healthy individuals, thereby reducing observable differences in the SpEn’s kurtosis. Moreover, both SP and HC may show similar adjustments in the vertical direction to maintain balance and stability. These adjustments could lead to similar mean SpEn values in the vertical direction. Moreover, SampEn evaluated on the Y and Z directions did not show statistically significant differences between the two groups. Also in this case, this could be due to compensatory mechanisms developed by SP to maintain the gait as normal as possible. Moreover, both SP and HC make adjustments to maintain balance and stability during walking. These adjustments can result in similar patterns of vertical and lateral heel movements, leading to comparable sample entropy values. Furthermore, SP may exhibit reduced gait variability due to cautious walking strategies to avoid falls, thus exhibiting more regular and predictable heel movements, resulting in SampEn values that do not differ significantly from those of HC. Additionally, it should be highlighted that the vertical and lateral components of heel movement might be less affected by stroke compared to other kinematic aspects. In fact, stroke primarily affects motor control and coordination, which might be more apparent in other directions or aspects of movement rather than in vertical or lateral movements. In this perspective, it is worth noting that all the EMG parameters selected by the MRMR exhibited significant differences between SP and HC. In fact, EMG signals measure the electrical activity produced by skeletal muscles, providing a direct window into muscle activation and neural control. In HC, the muscle activation is typically regular and coordinate, whereas in SP, muscle activation patterns could be irregular and unpredictable. However, SP often employ compensatory muscle activation strategies to overcome their motor impairments. These compensatory mechanisms involve recruiting additional muscles or using altered activation patterns to achieve desired movements. Moreover, stroke can disrupt the coordinated activation of muscle groups, known as muscle synergies, leading to abnormal EMG patterns, and stroke-induced pathophysiological changes in muscle properties, such as spasticity or altered muscle tone, can alter the complexity of EMG signals. Importantly, the multiscale approach revealed statistically significant differences between SP and HC at different temporal scales, demonstrating the necessity to further analyze the disruption of the muscle activation and gait patterns in SP using longer temporal series to evaluate more coarse-grained series, hence investigating more temporal series.

Notably, the CondEn metrics identified by the feature selection procedure exhibit strong differences between the two groups. This finding highlights the motor control disruption in SP. In fact, CondEn measures the uncertainty in one signal (kinematic) given knowledge of another (EMG), providing insights into the coupling and coordination between muscle activity and movement. These differences highlight the disrupted neuromuscular control in SP, and underscore the impact of stroke on the complex interplay between muscle activity and movement, making it a valuable metric for distinguishing between the two groups.

In order to assess statistical differences between the performance reached by the models fed with the different features set considered (i.e., unimodal kinematic, unimodal EMG, and multimodal kinematic + EMG), a DeLong test was conducted. As reported in Table 3, notable statistically significant disparities were detected between models fed with the kinematic feature set and those fed with the EMG feature set. Similarly, distinctions were observed between models utilizing the kinematic feature set alone and those incorporating kinematic + EMG features, demonstrating statistically significant differences. Conversely, no statistically significant differences were identified between models utilizing the EMG feature set exclusively and those integrating kinematic + EMG features. Consequently, the findings indicated that the inclusion of kinematic complexity metrics does not enhance the discriminatory capacity when combined with EMG entropy features. These findings are in line with previous studies, which demonstrated higher performance of EMG in assessing motor dysfunction in stroke patients, also employing complexity metrics [44,45,46,47]. Notably, in the literature, great attention is devoted to the employment of kinematic and EMG signals to evaluate the motor functions, but the number of studies investigating their combination is scarce. Particularly, most of the studies investigating the combination of these two techniques focuses on robotic rehabilitation on upper limbs [44,45,46,47]. To the best of our knowledge, this is the first attempt to combine stereophotogrammetry and EMG using complexity metrics for gait analysis of SP. This method underscores the efficacy of utilizing EMG-based features in the classification of post-stroke motor disorders through ML methodologies. Additionally, such an approach not only solidifies the classification process but also mitigates computational cost, thus further enhancing its practicality and efficiency. 

However, it should be noted that when conducting analyses on the identification of SP through EMG and kinematics during gait, it is crucial to consider the advantages of a multimodal strategy, even if one method, such as EMG, demonstrates superior performance. In fact, the integration of multiple data sources enhances the robustness and reliability of the diagnostic process. Utilizing diverse information modalities helps mitigate errors that might arise from relying on a single method. For instance, if EMG data are affected by noise or artifacts, kinematic data can provide an additional reference point to ensure accuracy. This redundancy of information from different modalities increases confidence in the obtained results. Moreover, multimodal data offer a comprehensive understanding of the patient’s gait. While EMG provides detailed insights into muscle activity, kinematic offers a complete view of gait biomechanics. Together, they present a holistic picture that is crucial to fully understand gait alterations in stroke patients. Furthermore, the integration of multimodal data facilitates personalized treatment plans. Detailed patient profiles derived from both EMG and kinematic data enable the customization of rehabilitation interventions. 

In fact, previous studies emphasized the advantages of employing multimodal approaches when dealing with SP. For instance, Murloy et al. used both kinematics and EMG parameters to cluster SP at the admission and after 6 months [48], whereas Barroso and colleagues found synergistic parameters between kinematic and muscle activation to discriminate the paretic and non-paretic sides of SP [49]. In addition, Saremi et al. investigated the validity of different accelerometry measurements in healthy and hemiparetic subjects [50].

Although our findings are promising, it is imperative to emphasize that additional investigations are warranted to validate these observations. The present findings may be attributed to the constraints posed by the limited sample size; therefore, it is essential to conduct further research, incorporating diverse datasets to corroborate these results. Moreover, the limited sample size did not allow to consider the hemorrhagic and ischemic patients separately. In this regard, it should be highlighted that previous studies have highlighted specific signs and symptoms that differentiate between hemorrhagic and ischemic strokes, including decreased consciousness [1], dilated pupils, agitation, severe headache, lower Glasgow Coma Scale (GCS) scores, seizures, and eye gaze impairments at higher rates than ischemic stroke patients [2]. Furthermore, the association of certain biomarkers with stroke types can provide insights into the underlying pathophysiology [3]. Concerning motor disabilities, hemorrhagic and ischemic stroke patients may both experience limb weakness, hemiparesis, spasticity, or ataxia [1,4], and ischemic strokes are associated with significant long-term disability and morbidity, impacting overall quality of life [5]. Hence, the evaluation of the difference between the two kinds of patients could benefit from a multimodal approach where the kinematic and EMG recordings can be integrated with also neuroimaging data coming from computerized tomography (CT) or magnetic resonance imaging (MRI) scans.

Furthermore, it is noteworthy that the dataset under consideration includes additional metrics, such as data derived from force platforms. Hence, comprehensive analyses encompassing these further information sources are indispensable for a more holistic understanding of the subject matter. Moreover, in this study, only the kinematic data of the calcaneus were considered; therefore, further studies are indeed necessary considering also other anatomical landmarks that can highlight changes in posture or coordination that may not be detectable through EMG alone, contributing to a more thorough diagnosis and monitoring of the rehabilitation process. To this end, it could be interesting to investigate the performance to assess gait dysfunction through the evaluation of complexity applied to low-cost kinematic systems based on video recordings in both the visible and the infrared spectrum, potentially providing further information on the muscle activation condition [51,52].

Additionally, forthcoming studies should involve regression analyses employing kinematic and EMG complexity features to assess clinical motor impairment scales (e.g., Performance Oriented Mobility Assessment (POMA) and Functional Ambulation Categories (FAC)), aiming to discern potential correlations between kinematic and EMG-derived complexity metrics and the severity of motor impairments assessed through established clinical scales. These findings could be a solid tool for healthcare professionals in clinical practice for assessing the effectiveness of the rehabilitation therapy in SP, but also for creating different gait models for various conditions affecting movement, especially those related to neurological issues. Finally, the employment of such models to signals acquired through wearable and printable EMG sensors in an Internet of Things (IoT) framework can provide a support for telemedicine’s applications by monitoring stroke patients during their daily activities. 

## 5. Conclusions

This study explored the potential of ML algorithms in the analysis of kinematic and EMG signals for the assessment of post-stroke patients. By incorporating a variety of classifiers and implementing a comprehensive set of complexity features derived from both kinematic and EMG signals, a robust tool for evaluating motor disabilities in stroke survivors was developed. The resulting models can be relevant for screening purposes. Further studies are indeed necessary to implement models able to provide a valuable support to physicians by delivering precise assessments of the motor impairments observed following a stroke.

## Figures and Tables

**Table 1:** Models’ performance assessed through accuracy, precision, recall, and area under the curve (AUC). The results are reported as mean value ± standard deviation.

Feature Set	Model	Accuracy (%)	Precision (%)	Recall (%)	AUC
Kinematic	Coarse Tree	86.68 ± 3.11	85.30 ± 3.85	87.87 ± 4.12	0.8812 ± 3.10
	Logistic Regression	84.08 ± 2.50	83.08 ± 2.26	84.98 ± 3.88	0.8360 ± 2.54
	Kernel Naive Bayes	90.26 ± 2.46	88.22 ± 3.43	92.10 ± 2.78	0.9090 ± 2.44
	Cubic SVM	88.24 ± 2.22	82.07 ± 2.18	93.74 ± 3.66	0.8810 ± 2.21
	Fine KNN	82.07 ± 3.13	77.44 ± 3.31	85.53 ± 4.35	0.8247 ± 3.09
	Bagged Trees	88.76 ± 2.82	86.88 ± 2.64	90.40 ± 4.06	0.8870 ± 2.82
	Medium NN	83.76 ± 2.84	82.81 ± 2.78	84.65 ± 4.52	0.8387 ± 2.85
EMG	Coarse Tree	98.72 ± 1.1 × 10^−16^	97.50 ± 2.2 × 10^−16^	99.94 ± 0.01	0.9870 ± 1.1 × 10^−16^
	Logistic Regression	99.71 ± 6.8 × 10^−3^	99.87 ± 7.8 × 10^−3^	99.56 ± 0.01	0.9869 ± 6.7 × 10^−3^
	Kernel Naive Bayes	98.54 ± 0.1	99.80 ± 3.5 × 10^−3^	97.38 ± 0.02	0.9853 ± 0.01
	Cubic SVM	99.85 ± 4.9 × 10^−3^	99.99 ± 0.01	99.71 ± 9.5 × 10^−3^	0.9992 ± 4.9 × 10^−3^
	Fine KNN	99.62 ± 6.2 × 10^−3^	99.92 ± 7.1 × 10^−3^	99.93 ± 0.01	0.9950 ± 6.3 × 10^−3^
	Bagged Trees	98.73 ± 1.8 × 10^−3^	97.49 ± 2.2 × 10^−16^	99.96 ± 3.5 × 10^−3^	0.9871 ± 1.9 × 10^−3^
	Medium NN	99.71 ± 6.5 × 10^−3^	99.95 ± 9.9 × 10^−3^	99.47 ± 9 × 10^−3^	0.9978 ± 6.5 × 10^−3^
Kinematic + EMG	Coarse Tree	97.86 ± 1.1 × 10^−2^	97.7 ± 1.4 × 10^−2^	98.01 ± 1.3 × 10^−2^	0.9775 ± 0.01
	Logistic Regression	98.28 ± 0.01	97.52 ± 1.8 × 10^−2^	99.04 ± 0.01	0.9842 ± 0.01
	Kernel Naive Bayes	98.95 ± 8.9 × 10^−2^	99.98 ± 0.01	98.01 ± 1.7 × 10^−2^	0.9890 ± 8.8 × 10^−2^
	Cubic SVM	98.98 ± 0.01	98.59 ± 1.7 × 10^−2^	99.38 ± 1.3 × 10^−2^	0.9898 ± 0.01
	Fine KNN	98.50 ± 1.2 × 10^−2^	97.78 ± 2 × 10^−2^	99.21 ± 1.3 × 10^−2^	0.9830 ± 1.1 × 10^−2^
	Bagged Trees	97.76 ± 0.01	97.44 ± 1.2 × 10^−2^	98.07 ± 1.4 × 10^−2^	0.9745 ± 0.01
	Medium NN	98.69 ± 0.01	97.92 ± 1.7 × 10^−2^	99.45 ± 1.3 × 10^−2^	0.9827 ± 1.1 × 10^−2^

**Table 2:** Comparison between SP and HC (unpaired *t*-test) for the selected gait, EMG, and CondEn features.

Metric	t Statistics	*p* Value
ApEn (EMG)	−8.679	4.5 × 10^−13^
ApEn X axis	−7.013	7.4 × 10^−10^
ApEn Y axis	−3.118	0.0026
ApEn Z axis	−6.733	2.5 × 10^−9^
SampEn (EMG)	−6.112	3.6 × 10^−8^
SampEn X axis	−6.233	2.2 × 10^−8^
SampEn Y axis	−1.578	0.1186
SampEn Z axis	−1.621	0.1090
FuzzyEn (EMG)	−15.502	1.6 × 10^−25^
FuzzyEn X axis	7.604	5.5 × 10^−11^
FuzzyEn Z axis	8.550	8.1 × 10^−13^
SpEn std (EMG)	−7.682	3.8 × 10^−11^
SpEn kurt (EMG)	−0.719	0.4739
SpEn skew (EMG)	−3.650	4.7 × 10^−4^
SpEn X axis mean	2.384	0.0195
SpEn X axis kurt	−0.561	0.5759
SpEn Y axis mean	−1.112	0.2694
Multiscale ApEn (n = 2)	25.823	5.7 × 10^−40^
Multiscale ApEn (n = 15)	−7.568	6.4 × 10^−11^
Multiscale ApEn (n = 16)	−7.621	5.1 × 10^−11^
Multiscale FuzzyEn (n = 5)	−17.210	2.8 × 10^−28^
Multiscale FuzzyEn (n = 8)	−17.807	3.3 × 10^−29^
Multiscale FuzzyEn (n = 19)	−19.149	3.3 × 10^−31^
CondEn EMG-X axis	19.802	3.7 × 10^−32^
CondEn EMG-Y axis	18.154	9.8 × 10^−30^
CondEn Z axis EMG	−10.873	2.7 × 10^−17^

**Table 3:** DeLong test results comparing the performances of the models fed using the different features set considered (i.e., kinematic, EMG, kinematic + EMG).

Model	Kinematic vs. EMG		Kinematic vs. Kinematic + EMG		EMG vs. Kinematic + EMG	
	z	*p*	z	*p*	z	*p*
Coarse Tree	−2.395	0.008	−2.112	0.0173	0.438	0.3305
Logistic Regression	−2.913	0.0017	−2.842	0.0022	0.139	0.4444
Kernel Naive Bayes	−1.967	0.025	−2.096	0.0180	−0.203	0.4194
Cubic SVM	−2.752	0.0030	−2.452	0.0070	0.784	0.2164
Fine KNN	−3.182	0.0007	−2.878	0.0019	0.707	0.2397
Bagged Trees	−2.342	0.0096	−2.210	0.0135	0.206	0.4182
Medium NN	−3.176	0.0007	−2.766	0.0028	0.944	0.1724

## Data Availability

Van Criekinge, Tamaya; Saeys, Wim; Truijen, Steven; Vereeck, Luc; Sloot, Lizeth; Hallemans, Ann (2023). A full-body motion capture gait dataset of 138 able-bodied adults across the life span and 50 stroke survivors. figshare. Collection. https://doi.org/10.6084/m9.figshare.c.6503791.v1 (accessed on 20 May 2024) [28].
